# Total Hip Arthroplasty Using the S-ROM-A Prosthesis for Anatomically Difficult Asian Patients

**DOI:** 10.1155/2015/690539

**Published:** 2015-10-25

**Authors:** Akira Hozumi, Kyousuke Kobayashi, Nobuhisa Tsuru, Chikara Miyamoto, Jyunichiro Maeda, Ko Chiba, Hisataka Goto, Makoto Osaki

**Affiliations:** Department of Orthopaedic Surgery, Nagasaki University Hospital, 1-7-1 Sakamoto, Nagasaki 852-8501, Japan

## Abstract

*Background*. The S-ROM-A prosthesis has been designed for the Asian proximal femur with a small deformed shape and narrow canal. In this study, the clinical and radiological results using the S-ROM-A prosthesis for Japanese patients with severe deformity due to dysplasia and excessive posterior pelvic tilt were examined. *Methods*. 94 hips were followed up for a mean of 55 months, with a mean age at surgery of 61 years. The primary diagnoses were 94 coxarthritis cases, including 51 dysplasia and 37 primary OA, 1 avascular necrosis, 2 traumatic arthritis, and 3 Perthes disease. Thirty-one hips had been treated with osteotomy of the hip joints. Preoperative intramedullary canal shapes were stovepipe in 23 hips, normal in 51 hips, and champagne-flute in 5 hips. The maximum pelvic inclination angle was 56°. *Results*. The mean JOA score improved from 46 points preoperatively to 80 points at final follow-up. On radiological evaluation of the fixation of the implants according to the Engh classification, 92 (97%) hips were classified as “bone ingrown fixation.” *Conclusion*. In primary THA, using the S-ROM-A prosthesis for Asian patients with proximal femoral deformity, even after osteotomy and with posterior pelvic tilt, provided good short- to midterm results.

## 1. Introduction

Total hip arthroplasty (THA) has excellent long-term outcomes with low rates of complications. However, it is difficult to treat patients with anatomic deformities of the hip joints, including developmental dysplasia of the hip (DDH), previous femoral and acetabular osteotomy, or severe posterior pelvic tilt with porotic bone. Treatment outcomes with cementless stems are inconsistent in these patients, and there is concern regarding the difficulties of treatment, such as frequent dislocation from early loosening or malpositioning of the implant used. In 1984, the S-ROM system (DePuy, Warsaw, IN) was developed as a stem for patients with these various types of anatomic deformities. This stem has a modular mechanism with a high degree of freedom. It consists of two parts, the sleeve and stem body. In addition, the stem and sleeve have various combinations and independent reaming in the proximal metaphyseal region of the femur and the diaphyseal region enables robust fixation with respect to various intramedullary canal shapes. Good results have been reported [1–4].

Compared with other races, Asian patients with hip diseases are of smaller stature, have less bone stock, and have a higher rate of DDH. Jingushi et al. [5] and Nakamura et al. [6] reported that more than 80% of primary diagnoses of osteoarthritis of the hip joints in Japan were related to DDH.

The S-ROM-A femoral prosthesis (DePuy) was developed in 2004 as a modification of the S-ROM based on the anatomical data of 270 Japanese hips. It has a shorter stem (5–25 mm shorter) with bullet tips, which reduce impingement with the femoral shaft and contribute to reducing the thigh pain and periprosthetic fracture. Furthermore, it has more neck offset options with a smaller diameter (changed from 11/13 to 9/10 neck taper) compared with the S-ROM femoral prosthesis.

It is important to select femoral stems that are suited for individual cases based on the patient's physique and anatomical and clinical state. For a case with severe deformities of the proximal femur, retroverted acetabulum, and posterior pelvic tilt in Japanese patients, we use the S-ROM-A prosthesis.

In this study, the clinical and radiological results using the S-ROM-A prosthesis for Japanese patients with severe posterior pelvic tilt, DDH with proximal femoral deformity, and postosteotomy hip joint were evaluated.

## 2. Materials and Method

### 2.1. Patients Status

A total of 115 primary cementless THAs using the S-ROM-A femoral component were performed in 120 patients by 5 hip surgeons between March 2005 and December 2011; the subjects were 89 patients (94 joints) who could be followed for at least 3 years and who had no rheumatoid arthritis or obvious metabolic bone disease such as renal osteodystrophy.

Ninety-four hips were followed up for a mean of 55 (range, 36 to 121) months, with a mean age at surgery of 61 (range, 42 to 84) years. There were 79 women and 10 men. Mean of patient height was 152 (range, 135 to 173) cm, of weight was 55 (range, 34 to 81) kg, and of body mass index was 23.4 (range, 17.2 to 34.9) kg/m^2^. The primary diagnoses were 94 coxarthritis (DDH: 51, primary OA: 37, traumatic arthritis: 2, avascular necrosis: 1, and Perthes disease: 3) including 33 postosteotomy hips. According to Crowe's classification [7], the degree of dislocation on preoperative X-rays was Group I in 53 hips, Group II in 25 hips, Group III in 12 hips, and Group IV in 4 hips.

The etiologies of the anatomical abnormalities of the hip are summarized in Table 1.

### 2.2. Surgical Procedure and Postoperative Protocol

Surgery was performed through a direct lateral approach in 82 hips, posterolateral approach in 6 hips, and subtrochanteric osteotomy in 6 hips. All operations were performed in a lateral position without trochanteric osteotomy. The acetabular component was used with a Pinnacle-A (DePuy, Warsaw, IN) in 73 hips, Duraloc in 15 hips (DePuy), and STD-CP (cemented cup, JMM, Osaka, Japan) in 6 hips. The average outer diameter of the cups was 50.1 (range, 44–56) mm. The inner head diameters were 22 mm in 1 hip, 26 mm in 8 hips, 28 mm in 51 hips, 32 mm in 30 hips, and 36 mm in 3 hips. The bearing surface was cobalt-chromium on polyethylene in 91 hips (cross-linked: 66, conventional: 25) and metal on metal in 3 hips.

The femoral diaphysis was reamed to the minor diameter of the stem. Hip joint stability was confirmed by the intraoperative dislocation test. When hip joint stability was insufficient, stem anteversion or cup anteversion was adjusted to achieve maximum stability of the hip joint. Subsequently, the final neck rotational angle against the sleeve was examined by the method similar to Kindsfater et al. [8]. Patients were allowed full weight-bearing on the day after surgery, except for subtrochanteric osteotomy cases.

### 2.3. Clinical and Radiographic Evaluations

Patients were examined preoperatively, postoperatively at 3 months and 6 months, and then yearly and at the final follow-up. Functional outcomes were evaluated using the Japanese Orthopaedic Association scoring system (JOA score), which has a total of 100 points, consisting of 40 points for pain, 20 for range of motion, 20 for ability to walk, and 20 for activities of daily living [9]. Preoperative morphology was assessed in terms of posterior pelvic tilt angle (PTA), femoral neck anteversion angle on CT scan, and the canal-flare index (CFI) [10], except in patients with some kind of osteotomy of the proximal femoral bone. PTA was defined as the angle formed by a vertical line drawn from the superior margin of the pubic symphysis and sacral promontory on lateral plain X-ray (Figure 2).

Stem fixation was classified as bony ingrowth, being fibrous stable, or being unstable according to the Engh classification [11–13]. Bone ingrowth was defined as apertures with no subsidence and no or less than 50% radiolucent lines around the sleeve. The presence of spot welds around the sleeve was considered bony ingrowth. Stable fibrous ingrowth was defined as a stem with no progressive subsidence and extensive radiolucent lines around the sleeve. Unstable fixation was defined as a stem with either progressive subsidence or migration and widely radiolucent lines around the sleeve. The stem position was classified as neutral, valgus, and varus according to 2 degrees more compared to the femoral shaft axis.

The intraoperative stem rotation angle against the sleeve was also examined [3, 8]. Intraoperative complications, such as femoral fracture, and postoperative complications, such as infection, dislocation, symptomatic pulmonary embolus, bone union of subtrochanteric osteotomy cases, and other implant-related complications such as thigh pain, were examined.

All radiological data were evaluated separately by three different surgeons (Akira Hozumi, Kyousuke Kobayashi, and Nobuhisa Tsuru).

In the statistical analysis, the Mann-Whitney* U* test was used to compare the difference in the mean JOA score between before operation and the latest follow-up, femoral anteversion angle, and CFI between DDH patients and primary OA patients. Statistical significance was established at *p* < 0.05.

## 3. Results

### 3.1. Clinical Outcome

The mean JOA score (pain/ROM/ambulation/ADL) improved from 46.1 points (14.0/11.7/8.1/12.3) preoperatively to 79.7 points (35.4/15.6/13.0/15.7) at final follow-up. In patients with a history of an osteotomy of the hip joint, the mean JOA score at final follow-up was 74.1 points. This was significantly lower than in patients without osteotomy of the hip joint (*p* < 0.05) (Tables 2(a)–2(c)).

There were 15 hip-related complications. A longitudinal calcar fracture around the sleeve in 9 hips and a greater trochanteric fracture in 6 hips occurred as intraoperative complications, and the CFI of these patients was relatively low (Table 3). Previous osteotomy did not affect intraoperative fracture (data not shown).

Thirteen cases were successfully treated by cerclage wiring procedures. Two cases of greater trochanteric fracture had nonunion.

Postoperative dislocation was not observed in any cases. Infection and symptomatic PE were not observed. Persistent groin pain after THA was observed in one hip with severe posterior pelvic tilt, which was diagnosed as iliopsoas impingement, but the patient preferred to continue with conservative management.

### 3.2. Preoperative Femoral Anteversion Angle, the Canal-Flare Index, and Posterior Pelvic Tilt

The femoral anteversion angle at the level of the osteotomy varied from −6° to 86°, with a mean of 28.4°. Mean posterior pelvic tilt was 20.6°. Femoral anteversion angle of DDH cases were significantly strong in comparison with the primary OA; adversely, the posterior pelvic tilt angle was significantly stronger in primary OA than DDH cases. According to preoperative CFI, there was no significant difference due to the primary diagnoses (Table 4). The preoperative CFI of femoral bone (without a history of osteotomy of the proximal femoral bone) was stovepipe in 23 hips, normal in 52 hips, and champagne-flute in 5 hips (Table 3).

### 3.3. Stem-Sleeve Rotation Angle

**Figure 1:**
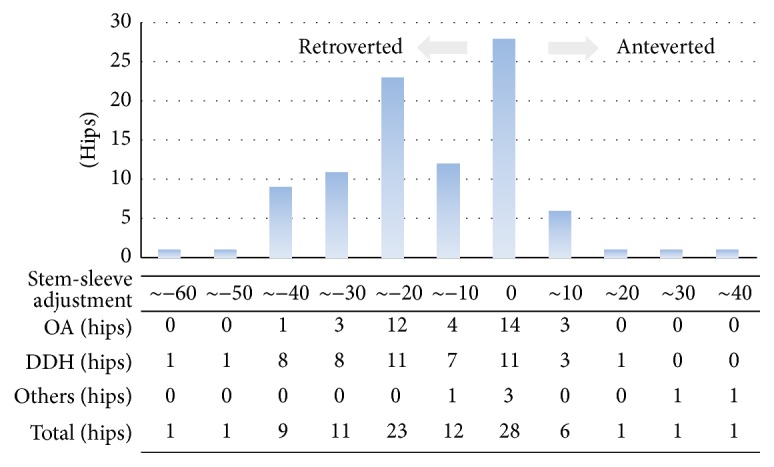
Adjustment of the rotation angle of the stem against the sleeve.

**Figure 2:**
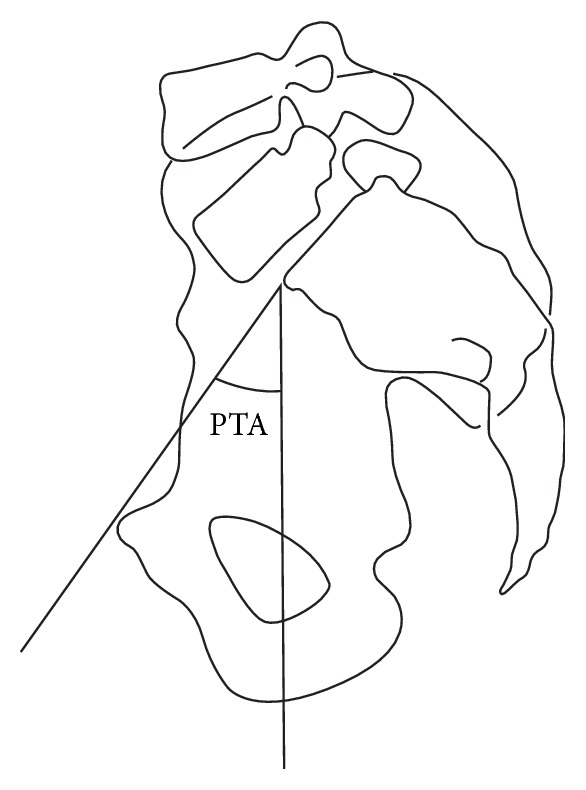
PTA was defined as the angle formed by a vertical line drawn from the superior margin of the pubic symphysis and sacral promontory on lateral plain X-ray.

Overall, femoral component version was changed in 67 hips (71%). The rotational angle of the stem ranged from 60° retroversion to 35° anteversion against the sleeve. The stem rotational angle against the sleeve according to preoperative diagnoses is shown in Figure 1.

### 3.4. Radiological Evaluation

Alignment of the stem was neutral in 88 hips (94%) and varus in 6 hips (6%). For fixation of implants according to the Engh classification, 91 hips (97%) were classified as “stable fixation.” None of the hips required revision surgery. Two hips were classified as “fibrous stable,” showing radiolucent lines around the sleeve, and one hip had unstable status. Spot welds around the sleeve were observed in 81 hips (88%). Second-degree stress shielding was observed in 57 hips (61%), and more than third-degree stress shielding was observed in 7 hips (7%). Stem subsidence was observed in 7 hips, including fibrous fixation cases, but it was not progressive except in the unstable case.

The overall radiological results according to the Noble classification, the filling rate of the distal part of the canal by the stem, and alignment of the stem are summarized in Table 5.

## 4. Discussion

The use of the S-ROM stem for primary THA in patients with DDH has been reported by Biant et al. [1]. They reported 0% aseptic loosening in 55 hips with anatomically difficult cases, of which 28 were severe DDH cases. Regarding the use of the S-ROM-A prosthesis, Kido et al. [2] and Tamegai et al. [3] reported that it was well-fitted for Asian DDH patients with short stature, a narrow canal, and severe proximal femoral deformity, showing favorable short- to midterm results. Zhao and Sun [4] reported good short-term results in elderly patients with poor bone quality containing Dorr type C using the S-ROM system. In the present study, stable bone ingrowth fixation was seen in 92 of 95 hips (96.8%). Furthermore, especially in stovepipe cases, all cases were stable fixation (Table 5). The reason for this depends on the fixation manner of S-ROM system. The S-ROM modular stem was designed to generate maximal contact between the metaphysis and diaphysis by independent reaming. Furthermore, proximal sleeve which has up to seven-size variation for each stem size and porous coated step surface generate good fit and fill and vertical stability, whereas, in the distal part, distal flute structure generates torsional stability and scratch fit. [14]. In particular, in the S-ROM A stem, it has a reduced stem length (5–25 mm shorter than the standard stem) and a bullet tip, which contribute to more fit and fill for the shorter Asian femur.

However, when large diameter stems are implanted, proximal stress shielding and thigh pain have sometimes been concerns [15, 16]. In the present series, no case had thigh pain at final follow-up. To explain this, we think that the hollow cylindrical stem with a coronal slot, shorter stem length, and bullet tip contribute greatly to decreasing the stiffness difference between the implant and the host bone, which is an important reason for postoperative thigh pain.

Second-degree stress shielding was radiographically detected in 57 hips (61%). Most of these occurred due to the formation of spot welds in the distal part of the sleeve, and this finding is consistent with trends seen in previous studies [17, 18]. Stress shielding of ≥3 degrees was observed in 7 hips (7%), of which 5 hips occurred in conjunction with some form of bone reaction to the distal part of the stem. The mean CFI in these patients was low at 2.2, and the stem had a high canal fill ratio or was varus inserted. However, bone ingrowth was ultimately achieved at the sleeve site for all patients, so these events were deemed to be temporary stress concentrations. In terms of their clinical course, patients tended to complain of femoral pain soon after surgery, but it resolved over time. However, in the case of excessive stovepipe and champagne-flute cases, careful implant selection and consideration of other stems (such as the well-documented cemented stem) should be considered because of the mismatch of fitting between the proximal and distal medulla even with this system.

Intraoperative fracture was seen in 15 hips (16%), higher than in previous reports [1–3]. The reason for this may have been that the mean CFI of this group was 2.97, indicating osteoporotic bone. In these cases, strong stress was added to the greater trochanter by the retractor in the Hardinge approach, and excessive reaming was done for large sleeve insertion. For these osteoporotic cases, preventive cerclage wiring should be done in the calcar portion, in addition to careful reaming.

Kindsfater et al. [8] reported that they needed stem-sleeve adjustment for 47.9% of cases, in which 79.3% of stems were anteverted. Tamegai et al. [3] reported that they needed anteversion for 18% and retroversion for 56% of their 196 DDH cases, and, furthermore, the postoperative dislocation rate was 0.9%. In the present study, stem-sleeve adjustment was needed in 71% (anteversion: 9%, retroversion: 61%), a higher rate, and there was no dislocation. We think that the Hardinge approach was one factor for the high rate of retroversion cases, and another factor was that the posterior pelvic tilt cases (≥30 degree) accounted for 23%. No postoperative dislocation was seen in the present study. In many past reports, the dislocation rate was around 1% [3, 8]. Many of these cases used a posterior approach. In the present study, a Hardinge approach, with which the short rotator can be preserved and good access to the acetabulum is possible for patients with strong anatomic deformities, was used. It is thought that this approach enables not only preservation of the short rotator but also accurate cup positioning, so that a high resistance to dislocation can be achieved.

**Figure 3:**
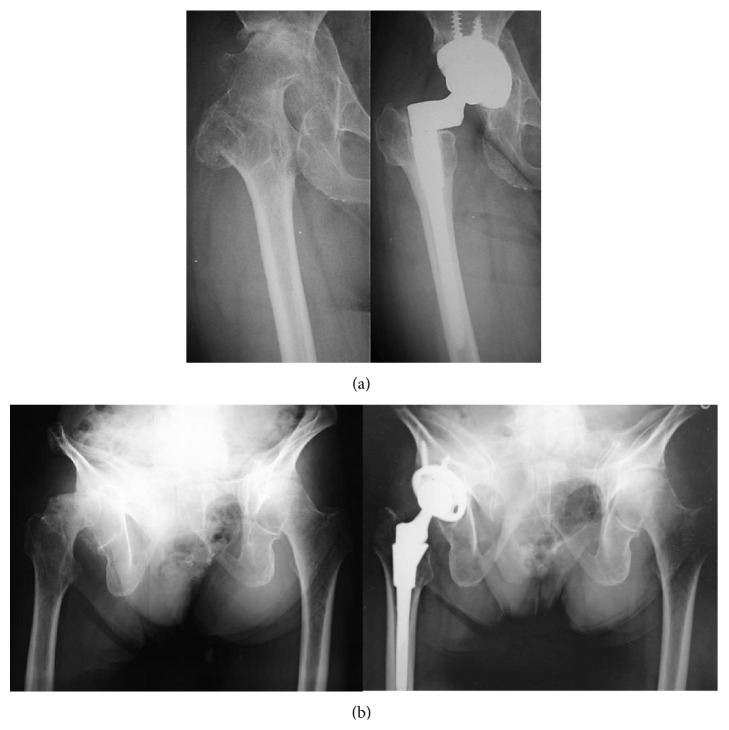
(a) 58-year-old female after pelvic and femoral osteotomy. Preoperative JOA was 52 points. Final follow-up (4Y) JOA was 74, stable fixation. (b) 84-year-old man with severe posterior pelvic tilt (50 degrees) 5 years after THA.

We used S-ROM-A proactively for THA in posterior pelvic tilt cases (Figure 3(b)). Change of pelvic inclination from lying position to standing position is frequently seen in aged patients. Furthermore, in these cases optimal adjustment of the acetabular cup is of utmost importance but is very difficult for ensuring stability and maximum range of motion of the prosthetic joint, without causing impingement on neck-liner or iliopsoas tendon. Thus, we think that it is advantageous for joint stability and prevention of implant-related complications to set the cup anatomically and to adjust rotation in the stem side as much as possible. Therefore, the use of S-ROM-A system is desirable for severe pelvic tilt cases.

In addition, this system is useful for patients with a high risk of dislocation with wide range of motion (alcoholic osteonecrosis, psychiatric disorders, paralytic disease, etc.) because of easy adjustment of stem version without removing the sleeve with bone ingrowth. On the other hand, there were several reports of catastrophic complications related to the S-ROM modular stem-sleeve junction [19–21]. In the present series, although there were no modularity-associated complications, such as fretting wear, corrosion, and breakage at the modular joint, longer-term follow-up is necessary. Furthermore, care is needed for young patients with high activity and severely obese patients when using the modular system.

Finally, in the present study, the JOA score at the final follow-up was relatively low (80 points) compared to previous reports [2, 3]. The high rate of patients who had postosteotomy hip joint likely affected the functional results.

## 5. Conclusions

Excellent short- to midterm results were obtained in anatomically difficult Asian patients with DDH, postosteotomy hip joints, and posterior pelvic tilt using the S-ROM-A system. The S-ROM-A prosthesis provides high stability of hip joints and reliable fixation for Asian patients.

## Figures and Tables

**Table 1:** Preoperative diagnoses and etiologies of anatomical abnormalities of the hip.

Preoperative diagnosis		Previous operation	
Primary osteoarthritis	37		

Developmental dysplasia	51	Rotational acetabular osteotomy	13
		Chiari and/or femoral osteotomy	14

Osteonecrosis	1	Sugioka's osteotomy	1

Perthes disease	3	Varus osteotomy	3

Traumatic arthritis	2	Osteosynthesis	2

Total	94		33

**Table:** (a) Preoperative and postoperative JOA score with history of osteotomy of the hip

	Preop	Postop
Pain	11.7	32.9
Range of motion	13.0	15.1
Walk	9.1	11.4
Activity of daily living	12.0	14.6
Total	45.8	74.1^**∗**^

**Table:** (b) Preoperative and postoperative JOA score without history of osteotomy of the hip

	Preop	Postop
Pain	14.9	36.4
Range of motion	11.1	16.0
Walk	7.7	13.6
Activity of daily living	12.4	16.1
Total	46.1	82.1^**∗**^

**Table:** (c) Preoperative and postoperative JOA score in all patients

	Preop	Postop
Pain	14.0	35.4
Range of motion	11.7	15.6
Walk	8.1	13.0
Activity of daily living	12.3	15.7
Total	46.1	79.7

^*∗*^ *P* < 0.05 (Mann-Whitney *U* test).

**Table 3:** The preoperative CFI of femoral bone and the number of intraoperative fractures.

Femoral bone shape (CFI)	Number of hips^*∗*^	Number of fractures (%)	Mean CFI of fracture patients
Stovepipe (<3.0)	23	7 (30.4%)	2.51
Normal (3.0–4.7)	51	8 (15.4%)	3.43
Champagne-flute (>4.7)	5	0 (0%)	—
Total	79	15	2.97

^*∗*^Number of hips without a history of osteotomy of the proximal femoral bone.

**Table 4:** Femoral anteversion angle, CFI, and pelvic tilt angle (PTA) according to preoperative diagnoses.

	Femoral anteversion	CFI	PTA
DDH	30.2 (17.6, −6–86)^*∗*^	3.56 (0.96, 1.62–6.2)	16.3, (8.1, −2.3–31.5)^*∗*^
Primary OA	21.5 (10.6, −1–42)^*∗*^	3.41 (0.89, 1.61–4.69)	27.3 (14.6, 14.1–55.6)^*∗*^
Others	23 (16.5, 2–48)	4.21 (1.24, 2.93–6.17)	26.35 (6.67, 17.05–32.9)
Total	28.4 (17.8, −6–86)	3.49 (0.97, 1.61–6.27)	20.59 (11.9, −2.3–55.6)

^*∗*^ *P* < 0.05 (Mann-Whitney *U* test).

**Table 5:** Radiological results according to femoral canal shape and implant positioning.

	*N*	Spot welding	Stress shielding (>III)	Bone ingrowth	Bone reaction around the distal part of the stem
Dorr classification					
Stove pipe	23	20	5	23	8
Normal	51	46	2	48	11
Champagne-flute	5	0	0	5	0
Postfemoral osteotomy	15	15	0	15	0
Filling rate of the distal part of the canal by the stem					
<80	10	7	1	8	4
80–90	40	38	4	40	6
90–100	44	39	2	43	7
Alignment of stem					
Neutral	88	79	6	85	15
Varus	6	6	1	6	4
Valgus	0	0	0	0	0
